# [Retracted] Long non‑coding RNA RNCR3 promotes glioma progression involving the Akt/GSK‑3β pathway

**DOI:** 10.3892/ol.2023.14055

**Published:** 2023-09-18

**Authors:** Bing Zhu, Shenyan Zhang, Ning Meng, He Zhang, Shaoji Yuan, Jin Zhang

Oncol Lett 18: 6315–6322, 2019; DOI: 10.3892/ol.2019.11002

Following the publication of this article, a concerned reader drew to the attention of the Editorial Office that the invasion assay data in Fig. 4 and the western blotting data shown in Fig. 5 on p. 6320 had already been featured in the following paper written by different authors at different research institutes [Shi M, Cao M, Song J, Liu Q, Li H, Meng F, Pan Z, Bai J and Zheng J: PinX1 inhibits the invasion and metastasis of human breast cancer via suppressing NF-κB/MMP-9 signaling pathway. Mol Cancer 14: 66, 2015].

Owing to the fact that the contentious data in Figs. 4 and 5 of the above article had already been published prior to its submission to *Oncology Reports*, the Editor has decided that this paper should be retracted from the Journal. The authors were asked for an explanation to account for these concerns, but the Editorial Office did not receive a reply. The Editor apologizes to the readership for any inconvenience caused.

